# Case Report: Dental autotransplantation for the resolution of odontogenic sinusitis using 3D replication

**DOI:** 10.3389/fdmed.2025.1607035

**Published:** 2025-06-13

**Authors:** Henry Paul Valverde Haro, Adriana Denisse Erazo Conde

**Affiliations:** ^1^ Program in Dentistry, National University of Chimborazo, Riobamba, Chimborazo, Ecuador; ^2^ Postgraduate Program in Dentistry, Universidad de los Hemisferios, Quito, Pichincha, Ecuador; ^3^ Postgraduate Program in Dentistry, Universidad Peruana Cayetano Heredia, Lima, Peru

**Keywords:** digital dentistry, tooth autotransplantation, donor tooth replica, sinusitis of dental etiology, autotransplanted tooth

## Abstract

This case report presents the use of dental autotransplantation, a procedure in which a donor tooth is transferred to a recipient site in the same patient, improving functional and esthetic characteristics. The advantages of this technique compared to conventional prostheses and surgical treatments, such as dental implants, are discussed. A 27-year-old patient with no relevant medical history was evaluated for pain and discomfort in fractured teeth with previous endodontic treatments and odontogenic sinusitis. A non-restorable maxillary first molar was diagnosed and extracted and replaced with a previously endodontically treated maxillary third molar, for which a 3D-printed replica was used to reshape the alveolar site. The intervention was successful, after which an adequate clinical and radiographic follow-up was carried out for 3 years, showing good bone formation and continuity of the periodontal ligament, with no signs of pathological resorption. This case demonstrates that, when properly planned and executed, dental autotransplantation can be an effective and biological alternative for dental rehabilitation, especially with the use of advanced technologies, such as cone beam computed tomography and 3D-printed replicas of donor teeth.

## Introduction

Dental autotransplantation involves transferring a donor tooth to another recipient site in the same patient, where its functional and esthetic characteristics may provide superior value compared to its initial position. This procedure can be used to replace anterior and posterior teeth (1, 2).

This therapeutic alternative increases the possibilities of dental rehabilitation in cases of missing or lost teeth, beyond the use of prostheses or conventional surgical treatments, such as dental implants (3).

Autotransplantation provides patients with a biological replacement alternative. It is an adaptable procedure with various clinical uses in patients of different ages. For example, autotransplantation of third molars has been used in adults to replace other permanent molars that were lost due to dental caries, periodontal disease, or endodontic infections (4, 5).

The success of this procedure and the achievement of better results depend on several factors, such as the general health of the patient, the particular tooth to be treated, the skill of the dental surgeon, and the respective postoperative care (6, 7). Long-term results of autotransplanted teeth have been mentioned in the literature with astounding longitudinal follow-ups of up to 26 years. According to some reports, autotransplanted teeth have a survival success rate between 75% and 98% (8). In addition, 5- to 10-year survival rates are greater than 95% in teeth with open apices (9).

When the criteria for choosing appropriate cases are met and the surgery is carried out properly, dental autotransplantation is very useful from a cost–benefit perspective, since if the apex is open, it is not necessary to carry out endodontic treatment (10). When the root is fully formed and endodontic treatment is carried out, the periodontal ligament and alveolar bone are preserved, unlike what happens with dental implants (11).

The criteria for selecting appropriate cases for dental autotransplantation, as outlined in the literature, include the presence of a suitable donor tooth, preferably with complete or appropriately staged root development to facilitate revascularization and continued root formation (4, 5), along with a recipient site with adequate alveolar bone volume, an absence of active infection or pathology, and a socket that can accommodate the donor tooth with minimal modification (3, 8). The patient's general health, compliance, and oral hygiene are also critical factors, as are surgical considerations, such as minimizing extraoral time and atraumatic tooth handling, to promote successful healing (12, 13).

This clinical report describes the guided autotransplantation of a fully rooted maxillary third molar into the reshaped alveolar site of a maxillary first molar that had been extracted because it was unsuitable for rehabilitation. The socket was modified to accommodate a 3D-printed replica of the donor tooth in the recipient area, which presented with odontogenic sinusitis.

## Case report

A 27-year-old female patient, with no relevant medical history, came to the office for an evaluation of teeth that were fractured 3 months previously. The patient reported pain when chewing and a bad odor and informed us that she had already undergone previous endodontic treatment. Table 1 shows the timeline of the relevant stages of guided dental autotransplantation treatment.

**Table 1:** Chronology of the relevant stages of the guided dental autotransplantation treatment.

Treatment sequence	Findings/treatment
Initial examination with periapical radiograph and 6 cm × 6 cm CBCT	Tooth 25 and 26 require root canal treatment Symptomatic apical periodontitis in tooth 25 Odontogenic sinusitis
Treatment of active pathology	Root canal retreatment and placement of a dental crown on tooth 25
Planning for dental autotransplantation	CBCT (Newton) Digital segmentation of tooth 28 (Exocad) 3D-printed replica of donor tooth (Prizma 3D, Bio Prov) Root canal treatment on tooth 28
Guided dental autotransplantation	Atraumatic extraction of tooth 26 The alveolus was modified with a No. 702 surgical drill Adaptation of the 3D replica of the donor tooth to the recipient site Occlusion tests with the 3D replica Atraumatic extraction of the donor tooth Splint with orthodontic wire to stabilize the donor tooth Control radiograph
Follow-up	20 days—removal of splint, control radiograph 3 months—control of reduced tooth mobility, no signs of inflammation 12 months—complete formation of periodontal space, normal mobility, control radiograph 24 months—no symptoms, normal appearance of periodontal space, control radiograph 36 months—no symptoms, normal appearance of periodontal space, no odontogenic sinusitis, control with CBCT

Upon intraoral clinical examination, the upper right first molar showed a positive response to vertical and horizontal percussion tests, with no positive response to thermal sensitivity tests. Furthermore, the upper right second premolar showed exposure of the gutta-percha and a coronal fracture, with similar responses to palpation and percussion tests.

**Figure 1:**
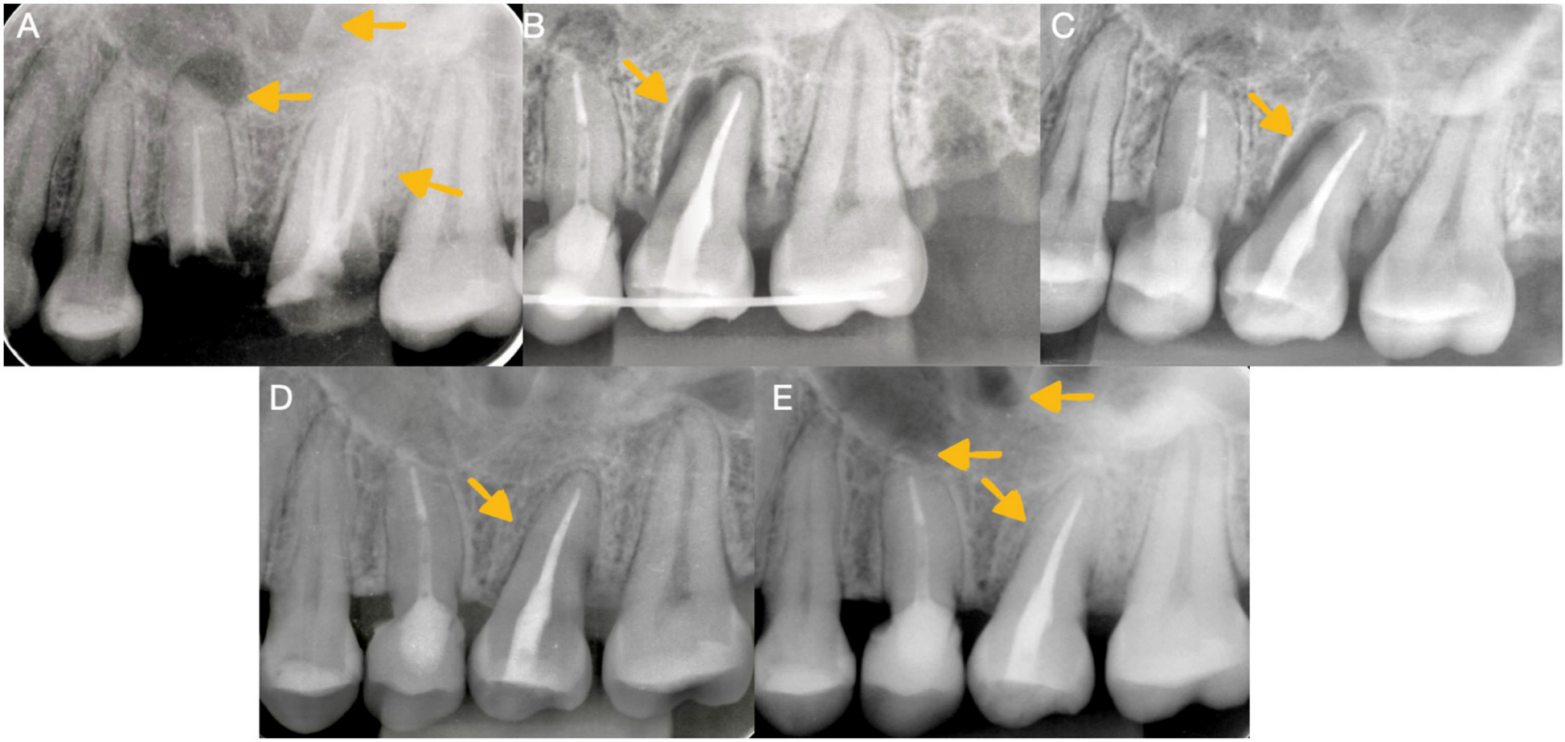
Radiographic imaging. **(A)** Initial radiograph of the first maxillary upper molar before retreatment and autotransplantation showing radiolucency in the right maxillary sinus. **(B)** Control radiograph of the autotransplanted third molar. **(C)** Radiographic image taken 1 month after autotransplantation. **(D)** Radiographic analysis conducted 1 year after autotransplantation revealed the presence of bone formation and the continuity of the periodontal ligament. **(E)** A radiographic analysis conducted 2 years after autotransplantation revealed the presence of bone formation and the continuity of the periodontal ligament.

In the initial periapical radiograph (Figure 1A), a deep caries invading the middle third of the distovestibular root was observed at the level of the right upper molar, and a fracture of the palatal, mesial, and distal walls was clinically observed. In addition to extensive caries at the level of the dental crown and inadequate obturation of the root canals, in the right upper premolar, a coronal fracture, exposure of the obturation material, and inadequate obturation of the canal with the presence of a periapical abscess were observed.

**Figure 2:**
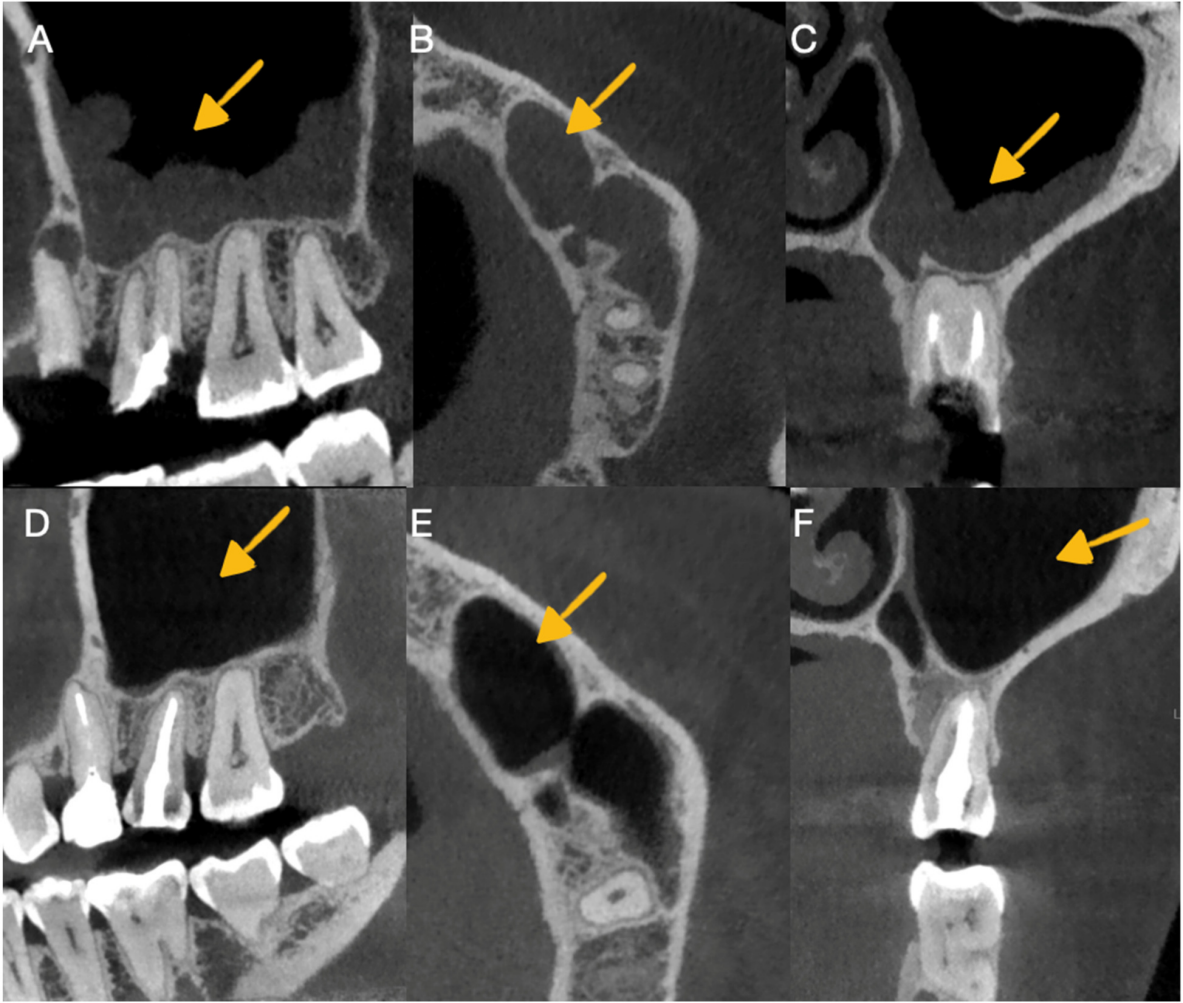
Imaging in CBCT of odontogenic sinusitis before and after autotransplantation. **(A)** Observation of sinusitis in the sagittal section before retreatment and autotransplantation. **(B)** Observation of sinusitis in the axial section before retreatment and autotransplantation. **(C)** Observation of sinusitis in the transaxial section before retreatment and autotransplantation. **(D)** Observation of sinusitis healing in the sagittal section. Periodontal ligament continuity and alveolar bone formation were observed in the autotransplanted tooth. **(E)** Observation of sinusitis healing in the axial section. **(F)** Observation of sinusitis healing in the transaxial section and control of the periodontal ligament continuity with alveolar bone formation in the autotransplanted tooth.

Following the guidelines of the European Society of Endodontics, cone beam computed tomography (CBCT) was performed with a small 6 cm × 6 cm field of view obtained from the tomographic equipment (GIANO HR, NewTon, Imola, Italy). The tomographic images confirmed the presence of apical periodontitis at the level of the right upper molar with subobturation of the root canal, and in the second premolar, a periapical abscess with subobturation was observed, which was associated with odontogenic sinusitis (Figures 2A–C).

**Figure 3:**
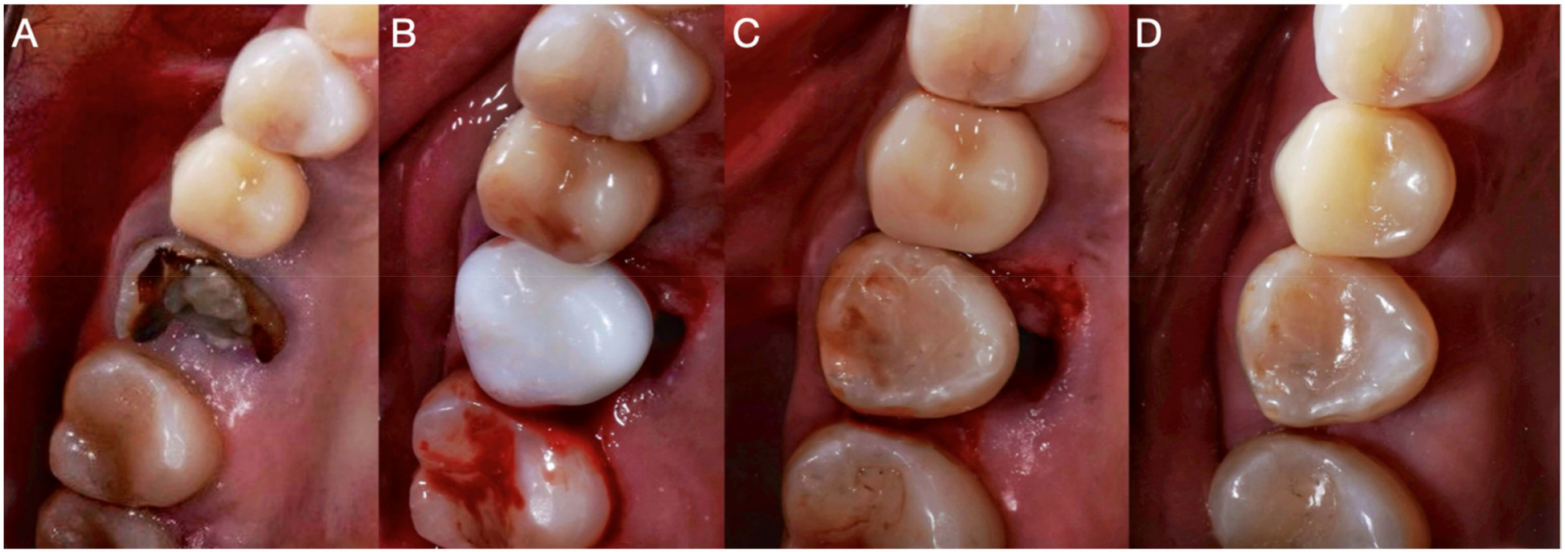
Occlusal intraoral photographs. **(A)** Initial photograph of the maxillary upper first molar. **(B)** Adaptation of the 3D replica in the prepared socket. **(C)** Adaptation of the autotransplanted third molar. **(D)** Control CBCT of the autotransplantation.

The initial treatment consisted of performing retreatment of the right upper second premolar, accompanied by placement of an intraradicular retainer and crown to restore form and masticatory function to the patient (Figure 3A), followed by autotransplantation of the right upper third molar into the recipient site of the right upper first molar. This treatment plan was considered a valid treatment option.

Using the requested CBCT, the root canal treatment of the donor tooth organ was performed in a single session to be able to perform the dental autotransplantation. The segmentation of the donor tooth was performed using Exocad DentalCAD 3.1 Rijeka (Darmstadt, Germany). The CBCT DICOM data were imported into Exocad, where the tooth of interest (tooth 28) was isolated using the software's dedicated segmentation tools. The process involved thresholding based on Hounsfield units to differentiate the tooth structure from the surrounding tissues, followed by manual refinement to accurately contour the root and crown. The finalized digital model was then exported as an STL file for further processing. The STL file was opened in Chitubox software (CTB Systems, Chitubox Basic, NJ, USA), where it was enlarged by 10% of the actual size of the donor tooth and then printed with biocompatible resin (Prizma 3D, Bio Prov, Sao Paulo, Brazil) using a three-dimensional printer (Anycubic Photon Mono 4K; Shenzhen, China). The replica was light-cured for 5 min, finished and polished, and then light-cured again for 10 min. The replica was then autoclaved for 5 min in a Tuttnauer autoclave at 135°C (Tuttnauer 2540, Beit Shemesh, Israel) while wrapped in gauze to avoid deformation.

**Figure 4:**
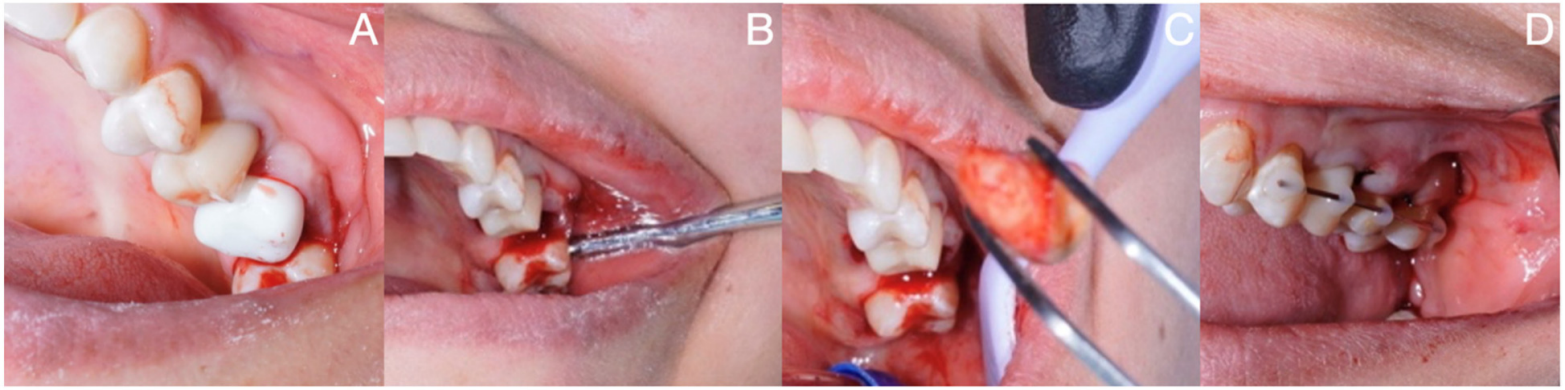
**(A)** 3D replica test in the alveolus of the maxillary right first molar. **(B)** Extraction of the maxillary third molar. **(C)** Visual inspection of the donor tooth. **(D)** Semi-rigid splint of the autotransplanted third molar.

During the surgical procedure appointment, infiltrative anesthesia with 2% lidocaine and a vasoconstrictor (Huons Co., Ltd., Cheonan, Republic of Korea) was applied at the level of the upper right molars to be operated on. First, the extraction of the right upper first molar was performed in an atraumatic way so as not to damage the adjacent soft tissues. Using a low surgical rotation drill (SS WHITE HP 702, Philadelphia, PA, USA), the alveolus was modified to adapt the three-dimensional replica to the dentally reshaped alveolar site (Figure 4A). Occlusion tests were performed with the replica inserted in the neoalveolar area, and the socket was modified until the patient did not feel any occlusal interference (Figure 3B).

Once the three-dimensional replica was adapted, the third molar was extracted atraumatically (Figure 4B) and immediately placed in the area of the reshaped alveolar site, which had been modified to receive the donor tooth (Figure 4C). An occlusion test was performed (Figure 3C), and the tooth was fixed with a rigid splint that was placed at the level of the two anterior premolars and fixed to the adjacent second molar (Figure 4D). Sterile gauze was placed in the area of the extracted third molar to generate hemostasis for 30 min. Regarding systemic medication, 875 mg of amoxicillin plus 125 mg of clavulanic acid (Curam 1,000 mg; Sandoz, Munich, Germany) once every 12 h for 7 days was prescribed and, as an analgesic, 120 mg of etoricoxib (Flogocox120; Bagó, Buenos Aires, Argentina) once daily for 4 days was prescribed, in addition to the post-surgical indications for a simple tooth extraction procedure.

The splint was kept in the mouth for 3 weeks (Figure 1B), after which it was carefully removed. The dental organ had a grade 2 mobility, so an appointment was made for an evaluation and control radiograph the week following the procedure. Subsequently, a decrease in mobility and healing of the adjacent tissues were observed 1 month after the procedure (Figure 1C).

The patient was monitored clinically and radiographically during the first and second years after the autotransplantation procedure (Figures 1D,E) and, in the third year, clinical (Figure 3D) and tomographic evaluations were performed, with adequate soft tissue healing and bone neoformation with continuity of the periodontal ligament at the level of the autotransplanted dental organ observed (Figures 2D–F).

## Discussion

Compared to other treatment options, autotransplantation of third molars exhibits physiological properties that make it an effective alternative to dental implants and fixed or removable prostheses (6). During the intervention, proprioception, vital periodontium, bone volume, occlusal resistance, and interdental papillae are preserved. When combined with the use of modern technology, autotransplantation can ensure a higher probability of clinical success with a favorable postoperative outcome (7).

Kadambari and Rajanikanth (14) stated that the extraoral time for the donor tooth should be less than 30 min and that during this period, continuous irrigation with saline solution should be performed to protect the periodontal ligament from dehydration; that is, the shorter the extraoral time and the more careful the handling of the tooth, the greater the preservation of the periodontal ligament cells. In our case report, the extraoral time was less than 1 min due to the previous adaptation of the 3D replica of the donor tooth to the alveolus, which had already been prepared as a recipient site.

The majority of published studies on autotransplantation include healthy patients (12, 15), which leads us to reflect on patient selection criteria and indicates that one should be cautious when applying this procedure to patients with systemic diseases. Further research is needed to explore the applicability of this technique in these population groups.

Kirmali et al. (16) pointed out that good initial stability, adaptation to the recipient alveolus, and stabilization with wires are important variables that significantly increase the rate of complete healing. Sutures are the first alternative for this type of procedure, with a total duration of use ranging from 7 to 90 days, but they show a low success rate. However, in recent years, the use of splints has been recommended for a period of 7–10 days (17). For this reason, in our study, the tooth was fixed with a semi-rigid splint for 20 days, which allowed functional movement, induced cellular activity in the periodontal ligament, and promoted bone regeneration (18).

Currently, the success rate of autotransplantation is high in the short and long term, even for mature teeth, where a statistically significant difference has been found in the success rate compared to teeth with an immature apex (11). Therefore, teeth with complete root formation can also be donor candidates without compromising the success of the technique, as demonstrated in the present clinical case (12).

Unlike a tooth with an incomplete root, autotransplantation of a mature tooth necessarily requires root canal treatment before or after the procedure; therefore, in the present case report, endodontic treatment was performed 3 days before autotransplantation surgery. However, it is better to use a fully formed root, as the length and thickness of the root wall are accurately known, which prevents root fractures during occlusal movement. This also allows for the precise adjustment of the amount of root resection, if necessary, for adaptation to the recipient alveolar cavity (19) or, as in the present case, modification of the recipient alveolus to avoid changing the root diameter.

The use of 3D-printed replicas has proven to be an important tool for increasing the success of autotransplantation, as accuracy in printing and replica design is crucial (20). The use of 3D-printed replicas was advocated by Kuo et al. (21) in their article on autotransplantation of teeth with mature apices. They reported a substantial improvement in treatment, as a 3D-printed replica allows for a reduction in extraoral time and damage to the donor tooth, mainly to the remaining root fibers of the periodontal ligament, by reducing manipulation to a minimum (9). Larger three-dimensional reproductions facilitate the handling and orientation of models, contributing to more efficient surgical planning and the construction of guides. However, it is essential to exercise rigorous control over this process, as excessive enlargement can distort or reduce the accuracy of the original structures (22).

The optimal duration of splint immobilization remains a subject of debate, and there is no consensus on the matter (23). Many authors use sutures or semi-rigid wires (5–7, 10, 17). Various sources recommend periods ranging from 7 to 14 days (9, 14, 19, 21, 23) or up to 2 or 3 weeks (24). The importance of this study lies in the fact that splinting was used for a period of 20 days, and because no signs of ankylosis or internal resorption were detected during follow-up. Stable continuity of the periodontal ligament was observed on both radiographs and CBCT scans. With regard to cost-effectiveness, autotransplantation is more cost-effective than implants and fixed partial dentures, as the planning and execution of the procedure can be performed in the endodontic clinic. The actual difference in cost-effectiveness between implants or dentures and autotransplantation may be even greater, as dentures often require additional fees for long-term maintenance (15).

Given the high success and survival rates of autotransplantation of teeth with mature apexes and its biological advantages, autotransplantation can be considered an alternative treatment option for adult patients if there is a suitable donor tooth, provided that proper planning and execution are carried out (25).

In the context of autotransplantation, platelet-rich fibrin (PRF) application may also decrease postoperative inflammation, accelerate new bone formation, and improve the periodontal vitality of the transplanted tooth (13). Although the preliminary evidence is promising, further research is required to establish standardized protocols and confirm these benefits definitively. In this case, PRF was not used, but good stabilization of the tooth in the reshaped alveolar site was achieved by means of a semi-rigid splint, resulting in adequate success after 3 years of follow-up.

Autotransplantation is a promising technique for preserving natural teeth; however, its long-term success can be compromised by various complications (19). The most prevalent of these are ankylosis, which affects alveolar growth in young patients; external resorption; and pulp necrosis, which requires timely endodontic treatment (10). Damage may also occur during the extraction and placement of teeth and in cases of occlusal misalignment. The prognosis is contingent upon the successful management of several pivotal factors, including the control of these elements, the extraoral time, and the employment of an appropriate surgical technique (20).

The case presented here provides unique evidence of how autotransplantation can be used as a treatment for odontogenic sinusitis, an aspect that is less frequently described. The autotransplantation procedure not only restored dental function but also helped resolve the sinus infection, which was evaluated during postoperative follow-up. However, it was not specifically treated by an otolaryngologist, demonstrating a dual therapeutic benefit. Although the results are promising, further studies with larger samples are needed to evaluate the procedure’s clinical efficacy.

The patient reported initial anxiety about losing a tooth and undergoing complex treatments. After a thorough explanation of the autotransplantation procedure, she felt reassured. Postoperatively, she expressed satisfaction with the recovery process and, at 3 years, reported excellent functional and esthetic outcomes, perceiving the transplanted tooth to be fully natural.

In conclusion, odontogenic sinusitis can be resolved with dental autotransplantation of a donor tooth after endodontic treatment, since the modification of the receptor alveolus allows for adequate adaptation of the 3D replica, thus eliminating the extraoral time of the donor tooth. With this procedure, we demonstrated that the periodontal ligament was present without pathological signs of external resorption or ankylosis of the donor tooth, with adequate alveolar bone formation. Although the procedure showed satisfactory results, further research and larger samples are needed to corroborate its applicability.

## Data Availability

The original contributions presented in the study are included in the article/Supplementary Material. Further inquiries can be directed to the corresponding author.
